# Identifying Quality Protein Maize Inbred Lines for Improved Nutritional Value of Maize in Southern Africa

**DOI:** 10.3390/foods11070898

**Published:** 2022-03-22

**Authors:** Isaac Amegbor, Angeline van Biljon, Nemera Shargie, Amsal Tarekegne, Maryke Labuschagne

**Affiliations:** 1 Department of Plant Sciences, University of the Free State, Bloemfontein 9300, South Africa; isaacamegbor@gmail.com (I.A.); avbiljon@ufs.ac.za (A.v.B.); 2 CSIR—Savanna Agricultural Research Institute, Tamale 00233, Ghana; 3 Agricultural Research Council—Grain Crops, Potchefstroom 2520, South Africa; shargien@arc.agric.za; 4 Zamseeds, 6483 Mugoti Road, Lusaka 10101, Zambia; a.tarekegne63@gmail.com

**Keywords:** maize inbred lines, nutritional value, protein quality

## Abstract

Malnutrition, as a result of deficiency in essential nutrients in cereal food products and consumption of a poorly balanced diet, is a major challenge facing millions of people in developing countries. However, developing maize inbred lines that are high yielding with enhanced nutritional traits for hybrid development remains a challenge. This study evaluated 40 inbred lines: 26 quality protein maize (QPM) lines, nine non-QPM lines, and five checks (three QPM lines and two non-QPM lines) in four optimum environments in Zimbabwe and South Africa. The objective of the study was to identify good-quality QPM inbred lines for future hybrid breeding efforts in order to increase the nutritional value of maize. The QPM lines had a lower protein content (7% lower) than that of the non-QPM lines but had 1.9 times more tryptophan and double the quality index. The lysine- and tryptophan-poor α-zein protein fraction was 41% lower in QPM than in non-QPM, with a subsequent increase in γ-zein. There was significant variation within the QPM inbred lines for all measured quality characteristics, indicating that the best lines can be selected from this material without a yield penalty. QPM lines that had both high protein and tryptophan levels, which can be used as parents for highly nutritious hybrids, were identified.

## 1. Introduction

Malnutrition, due to lack of a balanced diet, has become a chronic disease in under-developed and developing countries, affecting about two billion people [1] and leading to about 45% child mortality among infants under the age of five years [2]. Consequently, malnutrition has become an international problem and has caused an 11% loss of annual gross domestic product (GDP) in Africa and in other developing countries [1]. Maize is the third most important staple cereal food crop in the world after wheat and rice; it contributes about 30% of food-calorie intake and is a source of protein for more than four billion people in 94 developing countries [3].

Maize kernels consist of 61 to 78% starch, 6 to 12% protein, and 3 to 6% fat [4,5]. Maize protein exists largely in the form of zein proteins, subdivided into β-, γ-, and α-zein fractions based on amino acid sequences [5,6], with α-zein being the most abundant fraction. The zein storage proteins are deficient in lysine and tryptophan, which are essential amino acids for human and other monogastric animals, thus negatively affecting the crop’s nutritional value [7,8]. This led to the development of quality protein maize (QPM) in the 1960s, by conventional breeding through the introgression of the *opaque2* gene, and later modifier genes to harden the endosperm, to improve the nutritional content of traditional maize varieties to combat malnutrition. QPM has double the amount of lysine and tryptophan of normal maize and can supply 70 to 80% of human protein requirements, while non-QPM genotypes can only supply a maximum of 46% [9,10]. Several studies have been conducted on QPM to determine the lysine and tryptophan content of maize grains [11,12]. It is difficult to combine high grain yield with high-quality protein content in elite maize varieties because these two characteristics are often negatively correlated [13], either because the grain yield is directly involved in the process of seed modification or because the modifier gene(s) could be tightly linked to those responsible for protein synthesis [14]. 

Maize parental lines are the fundamental genetic materials essential for understanding the mechanism and principles of breeding [15,16]. In recent years, a large number of QPM inbred lines have been developed and successfully used as parents for generating hybrids and synthetic varieties [17,18]. Parental materials form the basis on which the development of stable and high yielding maize hybrids revolve [19]. As such, inbred lines should be extensively evaluated at different locations for consistency in the performance of traits of interest. Examining genotypes by environment interaction (GEI) is very important in crop development because several factors contribute to the performance of a genotype, since the production environment is variable [16]. The aim of this study is to assess the seed composition, the nutritional value and zein protein composition, and the yield performance of the new QPM inbred lines developed by the International Maize and Wheat Improvement Centre (CIMMYT) in order to identify the best lines to use as parents in developing high yielding and nutritious hybrids.

## 2. Materials and Methods

### 2.1. Genetic Material and Field Evaluation

The genetic material used consisted of 26 QPM inbred lines and nine non-QPM lines, as well as five commercial checks (three QPM and two non-QPM) (Table 1), developed by CIMMYT, Zimbabwe. The experiment was carried out under optimum conditions at Cedara (latitude −29.54°, longitude 30.26°, with an elevation of 1066 m above sea level, with reddish brown clay soils) and Potchefstroom (latitude −26.73°, longitude 27.08°, with an elevation of 1349 m above sea level, with brown sandy loam soils) in South Africa. All the trial locations are in summer rainfall regions, with December, January, and February receiving the highest rainfall. Average annual rainfall is 824 mm (Cedara) and 600 mm (Potchefstroom). The maximum temperatures during the maize growing season (October to April) vary between 22–25 °C (Cedara) and 24–29 °C (Potchefstroom). Trials in Zimbabwe were grown at CIMMYT, Harare (latitude 17°46′, longitude 31°02′, with an elevation of 1406 m above sea level), and Gwebi Agricultural College (latitude 17°13′, longitude 31° E, with an altitude of 1406 m above sea level). Gwebi is 27 km from Harare and has a similar climate. The annual rainfall of Harare is 845 mm with maximum temperatures of 25–31 °C in the growing season. All trials were conducted under natural rainfall without supplementary irrigation.

The experimental design used was a 5 × 8 randomised complete block design with two replications at each location. The experimental unit consisted of one-row plots, each 4 m long with inter-row spacing of 0.75 m and spacing within rows of 0.25 m. Two seeds were planted per hill, and seedlings were thinned to one plant per hill at four weeks after emergence to give a final plant population density of about 53,333 plants ha^−1^.

At Potchefstroom, the fertiliser regime consisted of compound fertiliser 3:2:1 (25) + Zn, applied as a basal application at planting, at a rate of 200 kg NPK (nitrogen, phosphorus, potassium) ha^−1^. LAN (Limestone Ammonium Nitrate) with 28% N was used for top-dressing in two equal splits at 28 and 56 days after emergence and at a rate of 100 kg ha^−1^ each. At Cedara, MAP (Monoammonium Phosphate) at 250 kg ha^−1^ was applied at planting, and LAN was given at 150 kg ha^−1^ in two equal splits of 75 kg ha^−1^ at 28 and 56 days after emergence. For the Zimbabwe trials, fertilisers were applied at the recommended rates of 250 kg ha^−1^ N, 83 kg ha^−1^ P, and 111 kg ha^−1^ K. Basal fertiliser application was conducted in the form of NPK, and an additional N application was conducted four weeks after seed emergence.

Yield was measured on a plot basis, but as the difference between the average QPM and non-QPM inbred lines was not significant, it was decided to focus only on quality characteristics in this study.

### 2.2. Seed Samples

The seed samples were obtained by self-pollinating two cobs from each entry in the two replications from all sites in order to prevent cross-pollination with unknown pollen, which could affect the quality characteristics. All the self-pollinated cobs were harvested and shelled manually and then bulked for each plot. The seed was placed in cold storage (5 °C) until laboratory analyses, which was carried out within a few weeks of harvesting.

### 2.3. Sample Preparation

One hundred kernels of uniform size were randomly selected from the bulked seed samples of each entry and replication; milled into flour using an IKA, A10 Yellowline grinder (Merck Chemicals Pty Ltd., Darmstadt, Germany); and then sieved with a 1 mm screen mesh.

### 2.4. Zein

Zein analysis was conducted using reverse-phase high-performance liquid chromatography (RP-HPLC). Zein extraction was adapted from the extraction method described previously [20]. Maize flour (0.20 g) was placed in 2 mL reaction tubes. An aqueous solution, containing 70% ethanol (Merck; 96% *v*/*v*), 24.5% filtered double distilled water, 5% beta-mercaptoethanol (Sigma; ≥99% *v*/*v*), and 0.5% sodium acetate (Saarchem AR; *w*/*v*), was prepared as a stock solution [21,22]. One millilitre of this solution was added to each tube. The mixture was agitated continuously on a vortex for ±16 h at ambient temperature, and the suspension was centrifuged for 15 min at 6000 revolutions per minute. The obtained supernatant was filtered through a 0.45 µm membrane filter using a syringe into glass vials, and it was then kept in a refrigerator at 4 °C until injection into the HPLC system.

RP-HPLC was performed on a Shimadzu Prominence LC System using a Jupiter C18 column (Phenomenex^®^) of 250 × 4.6 mm, with a 5 µm particle size and 300 Å pore size. Samples of 50 µL were injected and eluted with the solvent at 1 mL per minute flow rate with a column temperature of 55 °C. The two solvents used were labelled A and B. Solvent A was made up of LiChrosolv Acetonitrile (Merck) containing 0.1% (*v*/*v*) HiPersolv trifluoroacetic acid (TFA) (VWR chemicals), and Solvent B was filtered with deionised water comprising 0.1% (*v*/*v*) TFA and was set to run for 75 min per sample. Zein fractions were determined in a chromatograph using Shimadzu Class-VP 6.14 SP1 software. Based on the distinct peak retention time, β-, γ-, and α-zeins were determined as a percentage of the total zein content.

### 2.5. Amylose Content

Maize flour was used to determine the amylose content [23]. Absolute ethanol (Merck; *v*/*v*) was diluted with distilled water to 95% concentration. A 40 g sodium hydroxide (NaOH, Merck; *w*/*v*) was dissolved in double distilled water to prepare a 1 L solution. Other solutions were acetic acid, prepared by adding 57.2 mL of glacial acetic acid (Merck, Mr = 60.05; density = 1.05 g per mL) to 1 L distilled water, and iodine solution, prepared by dissolving 0.2 g iodine and 2.0 g potassium iodide in 100 mL distilled water.

A solution containing 1 ml of 95% ethanol (*v*/*v*) and 9 mL 1 M NaOH was added to each flour sample (0.1 g) in a 100 mL volumetric flask and vortexed. The samples were incubated and placed in boiling water to gelatinise the starch for 30 min before being allowed to cool for 1 h at room temperature, and they were then centrifuged for 5 min at 3000 rpm. A 100 μL sample was transferred into each 15 mL test tube, and 20 μL 1 M acetic acid and the prepared 200 μL iodine solutions were added. The final volume was made up to 10 mL using double distilled water, thoroughly mixed, and left to stand for 20 min for colour development to take place, before reading the absorbance at 620 nm on a UV–Vis spectrophotometer (Jenway Spectrophotometer Model 7315, UK, ST15 OSA).

The amylose percentage was calculated as follows:

$$\mathrm{Amylose}\;\left(\%\right)=\frac{\mathrm{Total}\;\mathrm{amylose}\;\mathrm{in}\;\mathrm{sample}\;\left(\mathrm{mg}\right)\;}{\mathrm{Sample}\;\mathrm{mass}\;\left(\mathrm{mg}\right)}\times 100$$

### 2.6. Tryptophan and Starch Content

Lysine and tryptophan levels are generally highly positively correlated in maize kernels [7,24], and, for this reason, only tryptophan was measured in this study. Flour samples (2 g) were defatted and analysed for tryptophan content, using a colorimetric method based on a glyoxylic acid reaction with the tryptophan present in the flour, in the presence of ferric chloride and sulphuric acid (H_2_SO_4_), according to the protocol of Nurit [24]. The optical density of tryptophan was read at 560 nm using a Jenway Spectrophotometer, Model 7315. The tryptophan percentage was then calculated [24]:

$$\mathrm{Tryptophan}\;\left(\%\right)=\frac{{\mathrm{OD}}_{60\mathrm{mm}}}{\mathrm{slope}}\times \frac{\mathrm{hydrolysis}\;\mathrm{volume}}{\mathrm{sample}\;\mathrm{weight}}\times 100$$

The total starch was determined using a polarimetric method [25]. Flour samples of 2.5 g were weighed into 100 mL Erlenmeyer flasks. Then, 50 ml of a 32% (*v*/*v*) GR HCL (Merck) solution was added, and the flask was placed in boiling water for 15 min, with the samples stirred every 5 min. The samples were taken out and allowed to cool to approximately 20 °C in a cool water bath. The mixture was then quantitatively transferred to a 100 mL volumetric flask. Then, 10 ml of a 4% (*w*/*v*) GR tungstophosphoric acid (Merck) was added to the solution, made up to 100 mL using double distilled water, and inverted several times. Double filtration with Whatman no. 4 filter paper followed until approximately 70 mL filtrate was collected. The filtrate was then read on an automatic polarimeter (ATAGO^®^ AP-300) at 589 nm.

The percentage of starch in the sample was computed as follows:

$$\mathrm{Starch}\;\left(\%\right)=\frac{10,000\times P}{L\times {\left[a\right]}_{D}^{20{}^{\circ}}\times S}$$

where *P* = the measured angle of the optical rotation in degrees; *L* = length (dm) of the sample tube, ${\left[a\right]}_{D}^{20{}^{\circ}}\;$= specific rotation of the pure starch, and *S* = exact mass of the sample.

### 2.7. Protein and Oil Content, and Quality Index

A 500 g sample of the self-pollinated seeds was used to determine the protein and oil contents (percentage weight basis, %wt) for each sample, using a near-infrared transmission spectroscopy (NIR) Perten Grain Analyzer (Model DA 7250, Perten, Instruments AB, Stockholm, Sweden), with three subsamples for each sample. The NIR calibration was confirmed with results from the wet chemistry of 50 samples before use. The quality index was calculated as the proportion of tryptophan to protein content.

### 2.8. Data Analysis

Analysis of variance was performed on the measured characteristics with Genstat (21st edition) [26] for the separate locations and across locations.

## 3. Results

### Analysis of Variance for Individual and Combined Locations

The effect of the inbreds was significant for all measured characteristics at all four locations and across locations, with the exceptions of protein content and β-zein content at Cedara and Gwebi (Table 2). Across all locations, the effect of the environment was highly significant (*p* > 0.01) for all characteristics. Genotype-by-environment interaction was highly significant (*p* > 0.01) for all characteristics except for β-zein, indicating that the inbreds did not rank the same across the different environments for the measured characteristics (Table 2).

On average, across the locations (Table 3), the protein content was significantly higher for non-QPM than for QPM, and the values ranged from 6.38% (QPM L6) to 9.78% (non-QPM L27). The oil content was similar for QPM and non-QPM, with the values ranging between 3.18% (QPM L30) and 5.65% (QPM L33). The starch content of QPM and non-QPM was similar, ranging from 60.20% (QPM L14) to 67.49% (non-QPM L28). As expected, the tryptophan content of QPM was much higher than that of non-QPM (1.9 times), with values ranging between 0.034% (non-QPM L36) and 0.091% (QPM L5 and L16). The quality index of QPM was double that of non-QPM, varying from 0.42 (non-QPM L39) to 1.73 (QPM L33). Amylose content was similar for QPM and non-QPM but varied extensively from 31.88% (non-QPM L39) to 53.18% (QPM L25). β-zein content was similar for QPM and non-QPM and varied from 2.35% (non-QPM L31) to 6.75% (QPM L31). There was a very large difference (41%) between QPM and non-QPM for γ-zein content, with the QPM values being the highest. The differences between the highest and lowest values were also very high (22.71% for non-QPM L20 and 70.62% for QPM L215). Likewise, the difference between QPM and non-QPM for α-zein content was 37% with non-QPM having the highest value and the values overall ranging from 26.68% (QPM L5) to 68.4% (non-QPM L20).

The values of significant correlations were low (Table 4), except for those for α- and γ-zeins, which were directly negatively correlated (r = −0.94). For the rest, only correlations of higher than 0.3 and *p* < 0.01 are mentioned. α-Zein content was negatively correlated with tryptophan content, while β-zein content was significantly negatively correlated with protein content. γ-Zein content was positively correlated with tryptophan content.

## 4. Discussion

With the development of QPM, it was reported that the protein content of QPM and that of non-QPM were similar [27], but in the inbred lines in this study, the protein content of the non-QPM was significantly higher (by 7%) than that of the QPM. Oil, starch, amylose, and β-zein were similar for the two groups of material, but there was a wide range of values for these characteristics, indicating that there are QPM lines with high protein content.

QPM contains the mutant gene *opaque* 2 (*o2*), which is responsible for the reduction in α-zeins, which contain no lysine or tryptophan, with an increase in non-zein proteins. Originally, this gene caused soft endosperm, which was overcome by using *o2* modifiers that ensured hard endosperm without the loss of the high lysine trait [28,29]. In this study, the tryptophan content of the QPM lines was 1.9 times that of non-QPM; this was also reflected in the QI, which was double in QPM compared to non-QPM. For a maize grain to be classified as a QPM, it must have a QI equal to or above 0.8 [30,31]. In *o2* maize, the zein fraction was found to be reduced by 50% [14], but it had higher contents of non-zein protein (albumin, globulin, and glutelin fractions), which are rich in lysine and tryptophan [32]. Therefore, the presence of the *o2* gene in the QPM lines would explain the large decrease in α-zeins in the QPM lines compared to non-QPM and the simultaneous increase in γ-zein in the QPM, where γ-zein was highly significantly correlated with tryptophan content. The highly significant negative correlation between α- and γ-zeins supports this finding. γ-Zeins are subdivided into three classes, namely, 16 kDa, 27 kDa, and 50 kDa. QPM lines were reported to have 2–3-fold higher levels of the 27 kDa γ-zein than non-QPM lines [32]. This may have caused the increase in the γ-zein fraction in the QPM lines. It was reported [33] that maize kernels with hard endosperm produced more α-zein (22 kDa and 19 kDa), while the intensity of these bands declined in QPM genotypes [19,33]. In the current study, the α-zein concentration was also much lower in QPM lines.

The significant genotype-by-environment interaction, observed for all the quality characteristics analysed except for β-zein, indicates that the environment plays an important role in the expression of these characteristics and protein fractions, changing the ranking of lines in different environments. Differences in amino acid contents, as a result of variations in soil nitrogen contents in different soybean varieties and different planting dates of buckwheat, were reported [34,35], demonstrating the environmental influences on protein composition, which was also the case in this study. This indicates that not only the line but also the environment influence the nutritional value of the grain.

Generally, the range of starch content values in the present study for the inbred lines is comparable to the values reported previously [36,37], with ample variation in all lines. Similarly, amylose, which is a major constituent of starch, varied significantly for the lines evaluated.

Some QPM lines, such as L16, L7, and L2, had a tryptophan content higher than 0.086 and could still maintain a protein content of 8.98, 8.54 and 8.44%, respectively. These lines can be used as parents in hybrid crosses in order to enhance the protein and tryptophan content in the seed.

## 5. Conclusions

The yield potential of the QPM and non-QPM inbred lines was similar (data not shown) but the QPM lines had much higher tryptophan and QI, and a much lower amount of the lysine- and tryptophan-poor α-zein fraction. Within the QPM lines, there was significant variation in protein content and α- and γ-zeins, as well as in starch and amylose content. The QPM lines had much better protein quality than that of the non-QPM lines, although the protein content was 7% lower on average. Some QPM lines were identified to have acceptable yield and to have both high protein quantity and quality; these can be used for future hybridisation to produce highly nutritious hybrids, which can be distributed to farmers in the region to address malnutrition caused by a maize-based diet.

## Figures and Tables

**Table 1:** Description of the QPM and non-QPM inbred lines and checks used in the study.

Code	Name	Donor
L1	CZL1330	QPM progeny
L2	CZL15041	QPM progeny
L3	CZL15055	QPM progeny
L4	CZL15073	QPM progeny
L5	CZL1471	QPM progeny
L6	TL135470	QPM progeny
L7	VL06378	QPM progeny
L8	TL155805	QPM progeny
L9	TL147078	QPM progeny
L10	TL147070	QPM progeny
L11	TL13609	QPM progeny
L12	TL145743	QPM progeny
L13	TL156614	QPM progeny
L14	CZL1477	QPM progeny
L15	CZL15074	QPM donor
L16	CZL0616	QPM progeny
L17	CZL083	QPM progeny
L18	CML572	Non-QPM parent
L19	EBL167787	Non-QPM check
L20	CZL0520	Non-QPM parent
L21	CZL99005	Non-QPM parent
L22	CML502	QPM donor
L23	CZL0920	QPM donor
L24	CML144	QPM donor
L25	CML159	QPM donor
L26	CML181	QPM donor
L27	CML197	Non-QPM parent
L28	CML312SR	Non-QPM parent
L29	CML488	Non-QPM parent
L30	CML491	QPM donor
L31	LH51	Non-QPM parent
L32	CZL00025	Non-QPM parent
L33	CZL15049	QPM tester
L34	CZL059	QPM tester
L35	CML444	Non-QPM tester
L36	CML395	Non-QPM tester
L37	CZL01005	QPM check
L38	CML511	QPM check
L39	CML312	Non-QPM check
L40	CZL1470	QPM check

**Table 2:** Analysis of variance of quality traits analysed for 40 inbred lines at four environments in Zimbabwe and South Africa during the 2017/2018 cropping season.

Source	Protein %	Oil %	Starch %	Tryptophan %	Amylose %	β-Zein	γ-Zein	α-Zein	QI
Cedara									
Rep	0.61	0.03	23.62 *	0.000003	15.77	54.90	14.30	110.80	0.03
Entry	0.86	1.44 *	9.59 *	0.000700 **	224.68 **	29.47	814.77 **	596.97 *	0.11 **
Error	0.59	0.47	4.81	0.000026	3.99	43.81	185.02	277.94	0.01
Potch									
Rep	0.01	0.82	5.61	0.00012 *	3.43	2.89	506.42	184.40	0.01
Entry	1.77 **	1.77 **	11.59 **	0.00007 **	127.90 **	2.56 *	626.25 **	749.91 **	0.11 **
Error	0.41	0.44	3.38	0.00001	5.62	1.09	173.98	102.07	0.01
Harare									
Rep	0.01	0.01	0.44	0.00005	25.97	51.47	265.39	1124.40 *	0.05
Entry	2.12 *	1.05 *	3.16 **	0.00048 **	2459.23	159.55 **	455.05	491.71 **	0.16 **
Error	0.45	0.08	0.13	0.00004	1861.53	31.43	279.22	132.02	0.01
Gwebi									
Rep	0.23	0.30	0.04	0.00005	23.99	34.95	8228.20 *	52.26	0.01
Entry	2.33	0.97 *	4.70	0.00050 **	166.79	11.12	390.22	591.20	0.11 **
Error	1.00	0.13	1.41	0.00002	7.54	23.68	454.75	806.43	0.01
Combined analysis									
Entry (G)	7.42 **	4.41 **	0.003 **	7.42 **	249.07 **	9.80 **	1490.51 **	1577.48 **	0.99 **
Environment (E)	592.77 **	4.14 **	0.008 **	592.77 **	823.45 **	67.80 **	976.73 **	606.62 **	20.71 **
GxE	1.03 **	0.88 **	0.000 **	1.03 **	232.70 **	2.21	213.41 **	209.52 **	0.21 **
Error	0.32	0.25	0.000	0.32	10.01	1.67	96.01	98.11	0.04

* *p* < 0.05, ** *p* < 0.01; QI = quality index.

**Table 3:** Means of quality traits for 40 inbred lines analysed across four environments in Zimbabwe and South Africa during the 2017/2018 cropping season.

Line	Line Status	Protein %	Oil %	Starch %	Tryptophan %	QI	Amylose %	β-Zein %	γ-Zein %	α-Zein %
L1	QPM	7.21	4.27	65.69	0.079	1.21	41.23	4.98	62.70	33.06
L2	QPM	8.44	3.94	66.35	0.086	1.11	44.24	4.33	46.49	49.26
L3	QPM	7.11	4.94	65.17	0.076	1.19	42.04	5.15	56.96	37.88
L4	QPM	7.54	3.78	66.84	0.068	1.01	45.92	6.00	35.27	58.66
L5	QPM	7.80	5.50	63.11	0.091	1.28	38.94	5.21	68.03	26.68
L6	QPM	6.38	3.83	66.72	0.079	1.68	46.56	4.79	44.18	38.19
L7	QPM	8.54	3.54	61.51	0.089	1.01	38.50	5.41	65.21	29.30
L8	QPM	9.43	5.09	63.25	0.076	0.88	46.10	5.92	40.08	54.05
L9	QPM	8.21	4.97	64.36	0.089	1.18	50.65	5.11	57.19	37.79
L10	QPM	9.64	6.31	61.14	0.076	0.83	37.30	5.81	37.52	56.67
L11	QPM	7.22	5.27	65.83	0.078	1.30	35.38	4.44	43.70	51.93
L12	QPM	7.13	3.62	66.91	0.078	1.12	36.07	6.75	62.61	30.56
L13	QPM	6.75	4.26	66.56	0.081	1.21	34.67	5.36	56.16	38.51
L14	QPM	8.63	4.15	60.20	0.083	1.01	33.75	5.48	63.42	31.02
L15	QPM	6.96	4.33	66.32	0.083	1.29	44.08	6.35	64.34	29.39
L16	QPM	8.98	5.06	63.61	0.091	1.07	44.12	4.71	49.60	45.69
L17	QPM	6.69	5.30	65.11	0.085	1.40	48.00	4.81	60.26	34.93
L18	non-QPM	7.81	3.47	66.20	0.035	0.51	39.82	6.18	44.82	48.92
L19	non-QPM	8.46	3.65	67.01	0.039	0.70	48.10	6.46	30.40	62.84
L20	non-QPM	8.17	4.50	65.06	0.044	0.71	49.42	4.55	22.71	72.83
L21	QPM	8.57	3.75	65.03	0.046	0.67	44.46	2.59	40.02	57.32
L22	QPM	8.00	4.57	63.45	0.081	1.56	37.23	5.38	51.73	42.90
L23	QPM	8.63	3.92	65.10	0.075	0.96	39.01	4.95	43.68	51.29
L24	QPM	8.24	4.12	65.50	0.085	1.15	43.61	5.71	64.69	29.60
L25	QPM	6.38	4.96	65.30	0.083	1.48	53.18	6.58	70.62	22.89
L26	QPM	8.53	3.86	65.64	0.075	1.06	39.37	3.06	43.13	53.81
L27	non-QPM	9.78	5.75	61.46	0.045	0.45	32.22	3.72	34.26	62.15
L28	non-QPM	7.09	4.07	67.49	0.038	0.65	36.04	5.64	32.26	62.10
L29	non-QPM	7.79	5.34	66.32	0.046	0.69	39.48	3.75	34.95	61.22
L30	QPM	7.95	3.18	66.14	0.043	0.58	44.74	6.62	57.88	35.43
L31	non-QPM	8.06	3.90	65.71	0.045	0.72	39.40	2.35	29.17	68.40
L32	non-QPM	9.30	4.09	63.85	0.040	0.46	31.32	3.69	30.40	65.99
L33	QPM	6.63	5.65	65.38	0.086	1.73	50.20	4.27	57.41	38.32
L34	QPM	6.53	5.64	65.01	0.075	1.30	45.43	4.58	64.01	31.49
L35	non-QPM	8.17	4.70	64.68	0.045	0.56	46.37	3.43	31.41	65.17
L36	non-QPM	7.16	4.77	66.42	0.034	0.59	31.88	6.85	29.98	63.16
L37	QPM	6.60	4.06	67.10	0.066	1.29	37.68	4.20	68.67	27.04
L38	QPM	7.53	5.04	66.19	0.079	1.38	39.50	5.41	47.62	44.80
L39	non-QPM	9.49	4.97	61.85	0.041	0.42	45.60	5.40	33.52	61.20
L40	QPM	6.77	4.86	66.10	0.076	1.36	44.49	4.93	55.98	39.08
QPM mean		7.69	4.54	64.99	0.078	1.18	42.29	5.13	54.45	39.92
Non-QPM mean		8.30	4.47	65.10	0.041	0.59	40.00	4.73	32.17	63.09
Total mean		7.86	4.52	65.02	0.068	1.02	41.68	5.02	48.33	46.29
LSD (0.05)		0.47	0.42	1.08	0.0051	0.16	2.62	1.07	8.11	8.20

QPM = quality protein maize; non-QPM = non quality protein maize; QI = quality index.

**Table 4:** Correlations between measured characteristics across all locations.

Characteristic 1	Characteristic 2	Correlation
α-Zein	γ-Zein	−0.94 **
	Tryptophan	−0.47 **
Amylose	Starch	0.18 *
β-Zein	Protein	−0.31 **
	Starch	0.15 *
Oil	Protein	0.22 **
	Tryptophan	0.19 *
γ-Zein	Tryptophan	0.41 **
Tryptophan	Starch	0.20 *

* *p* < 0.01, ** *p* < 0.001.

## Data Availability

Data are available from the corresponding author.
